# Changes of the bacterial composition in duodenal fluid from patients with liver cirrhosis and molecular bacterascites

**DOI:** 10.1038/s41598-023-49505-3

**Published:** 2023-12-27

**Authors:** Jim Höppner, Sandra Krohn, Ellen H. A. van den Munckhof, René Kallies, Adam Herber, Katharina Zeller, Jan Tünnemann, Madlen Matz-Soja, Antonis Chatzinotas, Stephan Böhm, Albrecht Hoffmeister, Thomas Berg, Cornelius Engelmann

**Affiliations:** 1 Division of Hepatology, Department of Medicine II, Leipzig University Medical Center, Leipzig, Germany; 2 DDL Diagnostic Laboratory, Rijswijk, The Netherlands; 3 Department of Environmental Microbiology, Helmholtz Centre for Environmental Research - UFZ, Leipzig, Germany; 4 Endocrinology and Nephrology, University Hospital Leipzig, Leipzig, Germany; 5 Division of Gastroenterology, Department of Medicine II, Leipzig University Medical Center, Leipzig, Germany; 6 Max von Pettenkofer-Institute for Hygiene and Clinical Microbiology, Ludwig Maximilians-University, Munich, Germany; 7 Institute for Liver and Digestive Health, Royal Free Campus, University College London, London, UK; 8 Department of Hepatology and Gastroenterology, Campus Virchow-Klinikum, Charité - Universitaetsmedizin Berlin, Berlin, Germany

**Keywords:** Liver cirrhosis, High-throughput screening

## Abstract

Small intestinal bacterial overgrowth and compositional changes of intestinal microbiota are pathomechanistic factors in liver cirrhosis leading to bacterial translocation and infectious complications. We analyzed the quantity and composition of duodenal bacterial DNA (bactDNA) in relation to bactDNA in blood and ascites of patients with liver cirrhosis. Duodenal fluid and corresponding blood and ascites samples from 103 patients with liver cirrhosis were collected. Non-liver disease patients (n = 22) served as controls. BactDNA was quantified by 16S-rRNA gene-based PCR. T-RFLP and 16S-rRNA amplicon sequencing were used to analyze bacterial composition. Duodenal bacterial diversity in cirrhosis was distinct to controls showing significantly higher abundances of *Streptococcus*, *Enterococcus* and *Veillonella*. Patients with bactDNA positive ascites revealed reduced spectrum of core microbiota with *Streptococcus* as key player of duodenal community and higher prevalence of *Granulicatella* proving presence of cirrhosis related intestinal dysbiosis. Regarding duodenal fluid bactDNA quantification, no significant differences were found between patients with cirrhosis and controls. Additionally, percentage of subjects with detectable bactDNA in blood did not differ between patients and controls. This study evaluated the diversity of bacterial DNA in different body specimens with potential implications on understanding how intestinal bacterial translocation may affect infectious complications in cirrhosis.

## Introduction

Spontaneous bacterial peritonitis (SBP) is the most common infectious complication in patients with liver cirrhosis and associated with a high one-year mortality^1^. Pathological bacterial translocation from the intestine into the mesenteric lymph nodes and systemic circulation is a key factor in the pathogenesis of SBP^2,3^. Accordingly, Llovet et al. could show in rats with cirrhosis and SBP that the degree of bacterial translocation was increased in comparison to healthy controls^4^. However, the intestine is a large organ with several structurally different parts and it remains unclear whether there is a predominant intestinal site from where bacterial translocation originates^2^. There is data suggesting that small intestinal bacterial overgrowth (SIBO) and ultrastructural abnormalities develop primarily in the duodenum^2,5,6^. SIBO defined by a bacterial quantity of > 10^5^ CFU/mL and/or intestinal dysbiosis defined by the presence of colonic bacteria in the upper jejunum^6^ can be detected in 35% to 68% of patients with cirrhosis compared to < 10% in healthy controls^7^. Even Bauer et al.^8^ could detect SIBO being present in 61% of patients with liver cirrhosis. Moreover, they found a close association between SIBO and the treatment with acid-suppressive therapy in cirrhotic patients. Increased small intestinal permeability, a leaky gut syndrome, develops along with SIBO as a consequence of altered expression of tight-junction proteins occludin and claudin-1 leading to the occurrence of bacterial DNA (bactDNA) in the blood of patients with progressing chronic liver disease^9,10^. In addition, pathogen-associated molecular patterns (PAMPs) such as Lipopolysaccharides (LPS) and other bacterial components translocate through the disrupted intestinal barrier into lymph nodes and blood vessels^11^ and endotoxemia is associated with SIBO^12^. SIBO was linked to the presence of activated CD33 + CD14 + macrophages in the intestine of patients with decompensated cirrhosis representing not only an early response to invading pathogens^13^ but also an indicator of mucosal repair processes and increased intestinal permeability^14^. Furthermore the clinical link between SIBO and (infectious) complication has been described previously^15^. Clear evidence that dysbiosis-induced leaky gut syndrome of the upper intestinal tract leads to bacterial translocation, with evidence of bacterial DNA in the blood and subsequently in the ascites, is still lacking in humans.

Therefore, we aimed at characterizing the duodenal, blood and ascites bacterial microbiome of patients with liver cirrhosis using a quantitative 16S rRNA gene based real-time PCR in combination with 16S rRNA gene amplicon sequencing.

## Results

### Patients’ characteristics

Duodenal fluid and blood samples from 103 patients with liver cirrhosis and 22 controls without liver disease were collected prospectively and analyzed retrospectively. Additionally, from 39 of 103 (37.9%) patients with liver cirrhosis corresponding ascites samples were obtained within 7 days after endoscopy during clinically indicated paracentesis.

Patients’ baseline characteristics, assessed at the time point of duodenal and blood sample collection, are shown in Table 1. Patients were mainly male in both cohorts [cirrhosis 76% compared to 59% in controls (p = 0.121)] and alcoholic liver disease was the predominant etiology of cirrhosis (72.8%; Table 1). Patients with cirrhosis were significantly younger than controls (mean age 60 vs. 70 years, p** < **0.001). At baseline, 32 (31.1%) patients with cirrhosis and 3 (13.6%) controls were under antibiotic treatment (p = 0.121). 30 (29.1%) patients with cirrhosis were receiving antibiotic therapy within 7 days before study inclusion whereas no control patient was pre-treated (p = 0.004).

**Table 1:** Patients’ characteristics at baseline.

Variable	Liver cirrhosis (n = 103)	Controls (n = 22)	Level of significance (p)
Age (years)	60 (29–80)	70 (37–88)	** < 0.001**
Gender (male/female), n (%)	78/25 (75.7%/23.3%)	13/9 (59.1%/40.9%)	0.121
BMI (kg/m^2^)	26.8 (19.5–45.8); n = 94	25.5 (18.6–34.7); n = 19	0.320
Aetiology of cirrhosis, n (%)			
Alcoholic	75 (72.8)	n.a	n.a
NASH	6 (5.8)		
Viral	7 (6.8)		
Autoimmune	4 (3.9)		
Cryptogenic	8 (7.8)		
Others	3 (2.9)		
Status of decompensation			
Bacterial infection	39 (37.9)	n.a	n.a
Ascites	72 (69.9)		
HE	33 (32.0)		
Bleeding	7 (6.8)		
MELD score, median (range)	14 (6–37), n = 94	n.a	n.a
Child pugh score, n (%)			
(n = 90)			
A	29 (32.2)	n.a	n.a
B	35 (38.9)		
C	26 (28.9)		
Ascites leukocyte count (cells/mm^3^)	166 (50–1779); n = 39	n.a	n.a
PMN (cells/mm^3^)	27 (10–480); n = 13		
SBP at baseline, n (%)	7/103 (6.8)	n.a	n.a
SBP 6 months prior	8/103 (7.7)		
Baseline, n (%)			
Bilirubin (μmol/L)	32.4 (4.4–530.1); n = 103	9.7 (5.7–179.4); n = 21	** < 0.001**
Albumin (g/L)	34.5 (17.0–54.6); n = 95	40.6 (18.5–48.5); n = 14	**0.014**
INR, median	1.3 (1.0–3.3); n = 92	1.1 (0.9–1.2); n = 17	** < 0.001**
Serum creatinine (μmol/L)	85 (6–588); n = 103	78 (47–132); n = 21	0.137
GFR (mL/min)	72.0 (7.5–149.7); n = 90	90.3 (41.0–101.5); n = 18	0.468
ALAT (µkat/L)	0.44 (0.12–2.16); n = 100	0.34 (0.18–6.79); n = 21	0.189
GGT (µkat/L)	1.74 (0.19–25.36); n = 101	0.76 (0.18–43.39); n = 21	**0.023**
White blood cell count (10E9/L)	6.5 (2.1–35.9); n = 101	7.0 (1.0–15.2); n = 22	0.681
Hemoglobin (mmol/L)	6.6 (4.1–10.3); n = 101	7.7 (4.4–9.6); n = 22	0.097
C-reactive protein (mg/L)	13.4 (0.3–146.1); n = 101	7.4 (0.3–201.5); n = 22	0.241
Antibiotic therapy; n (%)			
–at baseline	32/103 (31.1)	3/22 (13.6)	0.121
–7 d before baseline	30/103 (29.1)	0/22	**0.004**
PPI, n (%)	66/103 (64.41)	16/22 (72.7)	0.473
NSBB, n (%)	60/103 (58.2 )	0/22	** < 0.001**
Lactulose, n (%)	49/103 (47.6)	0/22	** < 0.001**
Esophageal varices, n (%)	84/103 (81.6)	0/22	** < 0.001**
History of esophageal varices bleeding, n (%)	20/103 (19.4)	0/22	**0.023**
HE at baseline, n (%)	37/103 (35.9)	0/22	** < 0.001**

Categorical data are displayed as absolute values and as percentage. Metric data are shown as median (range). Groups were compared using either Mann–Whitney *U* test or Chi-square test.

*ALAT* alanine-amino-transferase, *GFR* glomerular filtration rate, *GGT* gamma-glutamyltransferase, *HE* hepatic encephalopathy, *INR* international normalized ratio, *MELD* model for end-stage liver disease, *NASH* non-alcoholic steatohepatitis, *NSBB* non-selective beta-blockers, *PMN* polymorphonuclear leukocyte count, *PPI* proton pump inhibitor, *SBP* spontaneous bacterial peritonitis, *n.a*. not applicable.

Significant values are in bold.

### Patients with cirrhosis did not demonstrate higher bactDNA quantities in duodenal fluid and blood compared to liver healthy controls

First, we analyzed the rate and quantity of bactDNA in different body specimens to understand whether cirrhosis is linked to a higher risk of bacterial dissemination.

In all duodenal fluid samples bactDNA was quantified with a similar concentration in healthy controls and in patients with cirrhosis (9.4 × 10^8^ copies mL^–1^, range: 8.3 × 10^4^–2.8 × 10^10^, versus 3.2 × 10^8^ copies mL^–1^, range: 8.1 × 10^3^–7.7 × 10^10^; p = 0.257). BactDNA was present in less than 10% of all blood samples without difference between cirrhosis and controls (6.8% versus 9.1%, p = 0.889). The median blood bactDNA level in the cirrhosis was 7.3 × 10^3^ copies mL^–1^, as compared to 1.4 × 10^4^ copies mL^–1^ in controls. *Staphylococcus* was identified in 5 out of 9 (55.5%) bactDNA positive blood samples representing the most frequently detected bacterial genus (Table S1). Seven ascites samples (17.9%) from patients with an ascites leukocyte count of < 500 cells/mm^3^ (range 90–350) were bactDNA positive with up to 5.6 × 10^3^ copies ml^–1^ (median: 9.7 × 10^2^ copies ml^–1^). None of the patients showed bactDNA simultaneously in ascites and blood samples.

### T-RFLP analyses did not reveal compositional changes of bacteria from duodenal fluid of cirrhosis and controls

**Figure 1:**
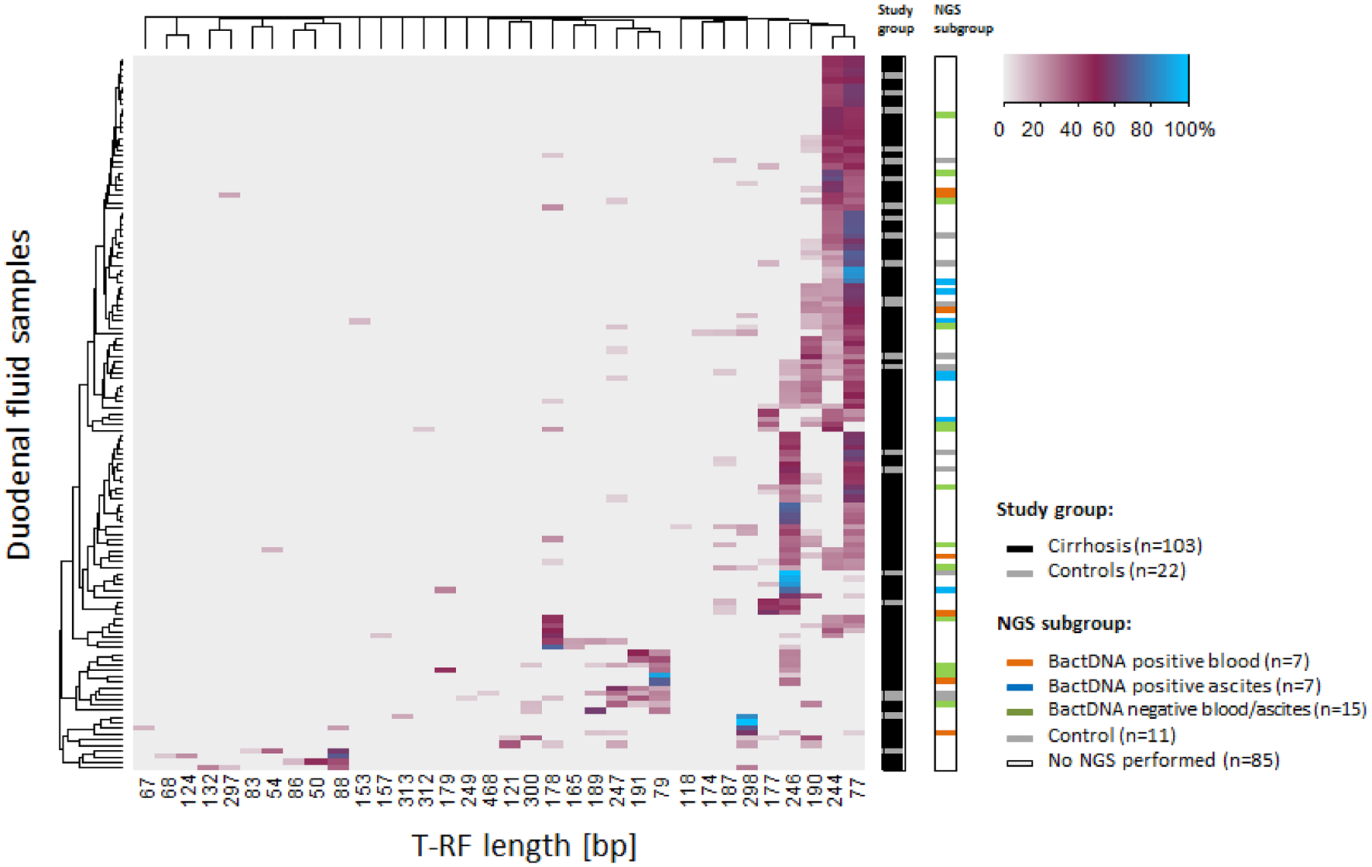
Two-way cluster analysis of bacterial DNA (bactDNA) from T-RFLP profiles performed in duodenal fluid samples. *MspI* was used as restriction endonuclease and Bray–Curtis index (deviation = 3) served for comparison. Duodenal samples (n = 125) are represented by rows, according to the study group color code on the right side of the graph (*black*: cirrhosis; *grey*: controls). A second legend presents the subgroup of duodenal samples that were analyzed by amplicon deep sequencing. It includes those samples whose paired blood was bactDNA-positive (*orange*; n = 7), whose paired ascites was bactDNA-positive (*blue*; n = 7), selective samples with bactDNA negative blood and ascites (*green*; n = 15) and controls (*grey*; n = 11). Duodenal samples are clustered according to the similarity of their T-RFLP profile. T-RF in base pairs are represented by columns and clustered by co-occurring T-RF within the sample. The color key (upper right corner) shows the relative abundance of the T-RF.

In order to determine the duodenal bacterial composition in relation to the presence or absence of systemic spread of bactDNA we performed terminal restriction fragment length polymorphisms (T-RFLP) analysis in all of the patients with cirrhosis (n = 103) and compared them to controls (n = 22). T-RFLP provided terminal restriction fragments (T-RF) with a length of 77 bp and/or 245/246 bp in the majority of duodenal samples (89.9%) (Fig. 1). Sequence analyses of cloned fragments showed that this T-RF could be assigned to *Streptococcus* and *Staphylococcus/Lactobacillus*, respectively. Overall, duodenal fluid showed a heterogeneous T-RF composition in both study cohorts and no significant differences in T-RF clusters between patients and controls were detected. However, alpha-diversity Shannon effective numbers from T-RFLP analysis revealed a significant higher bacterial diversity in patients with cirrhosis compared to controls (Wilcoxon-Mann–Whitney-Test; p = 0.01327). Median of Shannon effective numbers was 3.865 (1.625–12.743) for cirrhosis patients and 3.246 (1.078–6.599) for controls. The homogeneity of T-RF in duodenal fluid was within the same range in both cohorts (median Evenness (range): E_cirrhosis_ = 0.577 (0.356–0.947) vs E_controls_ = 0.563 (0.462–0.985); p = 0.853).

### 16S rRNA gene amplicon sequencing revealed a greater interindividual variability of bacterial diversity in duodenal fluid of patients with cirrhosis and bacterial DNA positive ascites

Since we detected almost no differences in the overall community composition between cirrhosis and controls using T-RFLP profiles we wanted to understand whether the presence of bactDNA in blood or ascites is linked to a distinct duodenal pathogen profile. For that purpose, we analyzed samples from three subgroups of patients with cirrhosis and compared them to controls by using 16S rRNA gene amplicon sequencing: (i) patients without bactDNA in blood and ascites (n = 15), (ii) patients with bactDNA positive blood samples (n = 7), (iii) patients with bactDNA positive ascites samples (n = 7), controls without liver disease (n = 11).

Results indicated that gene amplicon sequencing resulted in sufficient sequencing depth in all duodenal fluid samples (n = 40; S2 Figure). In total, 2390 amplicon sequence variants (ASVs) were identified in duodenal fluid samples. 1557 ASVs were detected in cirrhosis and 724 ASVs in controls representing 134 and 105 genera, respectively. Absolute abundance of the 20 most abundant genera in duodenum varied between both study groups and in individuals within each cohort (S3 Figure). Similar to clone analyses corresponding to T-RF lengths, *Streptococcus* was the most abundant bacterial genus in both groups followed by *Rothia* and *Veillonella* (S3 Figure).

Alpha-diversity analyzes, i.e. Shannon diversity, Pielou’s evenness and richness did not reveal differences between cirrhosis and controls (Fig. S4A). In addition, no significant differences in alpha diversity metrics were identified in patients with or without corresponding bactDNA detection in blood or ascites (Fig. S4B), respectively.

**Figure 2:**
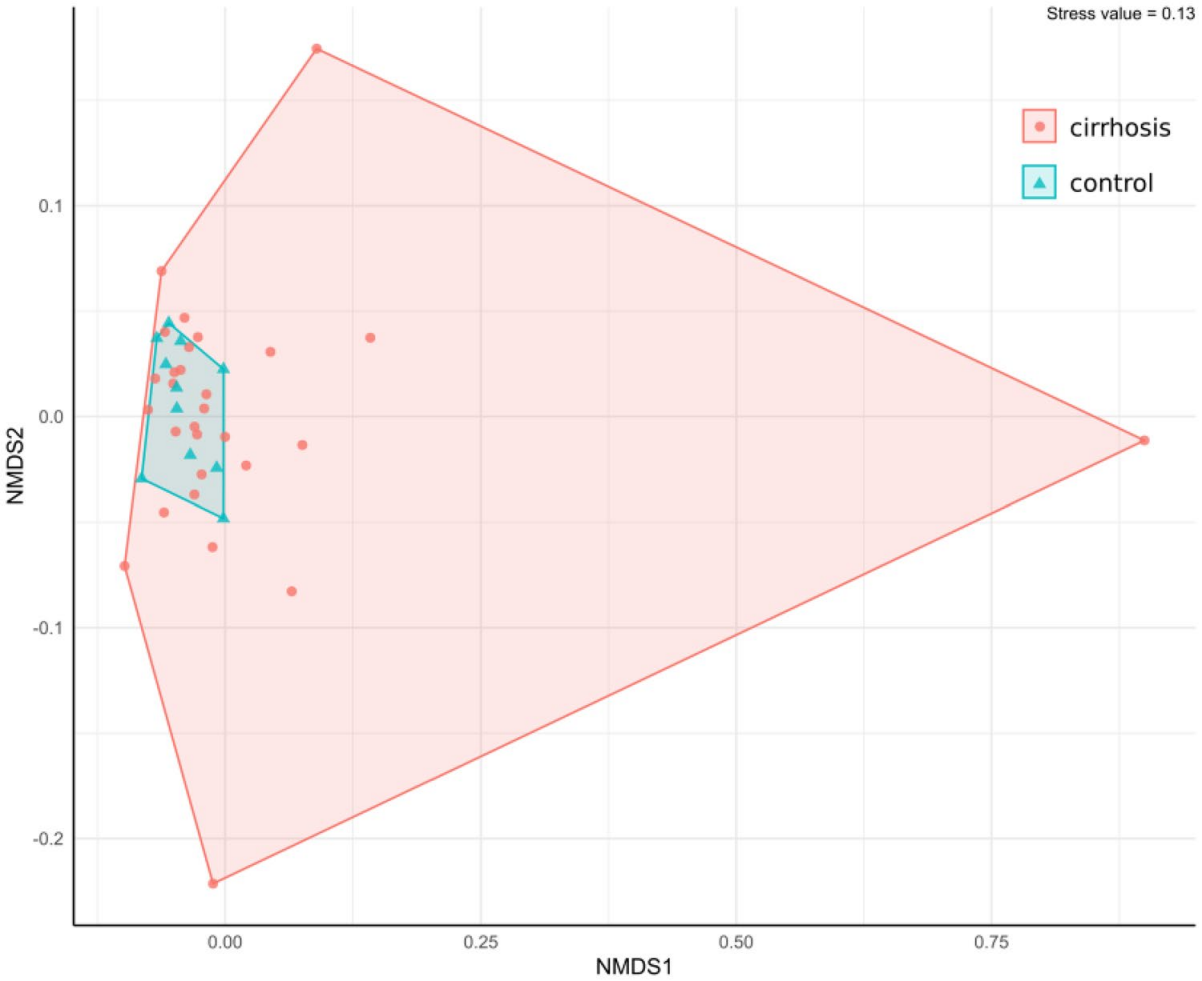
Nonmetric multidimensional scaling (NMDS) plot showing variances in bacterial composition of duodenal fluid samples from cirrhosis and controls.

As shown in Fig. 2 the bacterial spectrum in duodenal fluid from control patients could be clustered closely together in contrast to the cirrhosis group indicating a greater similarity of duodenal bacterial microbiome composition among controls as compared to patients with cirrhosis.

### Patients with bactDNA positive ascites showed a higher prevalence of Granulicatella in duodenal fluid

**Figure 3:**
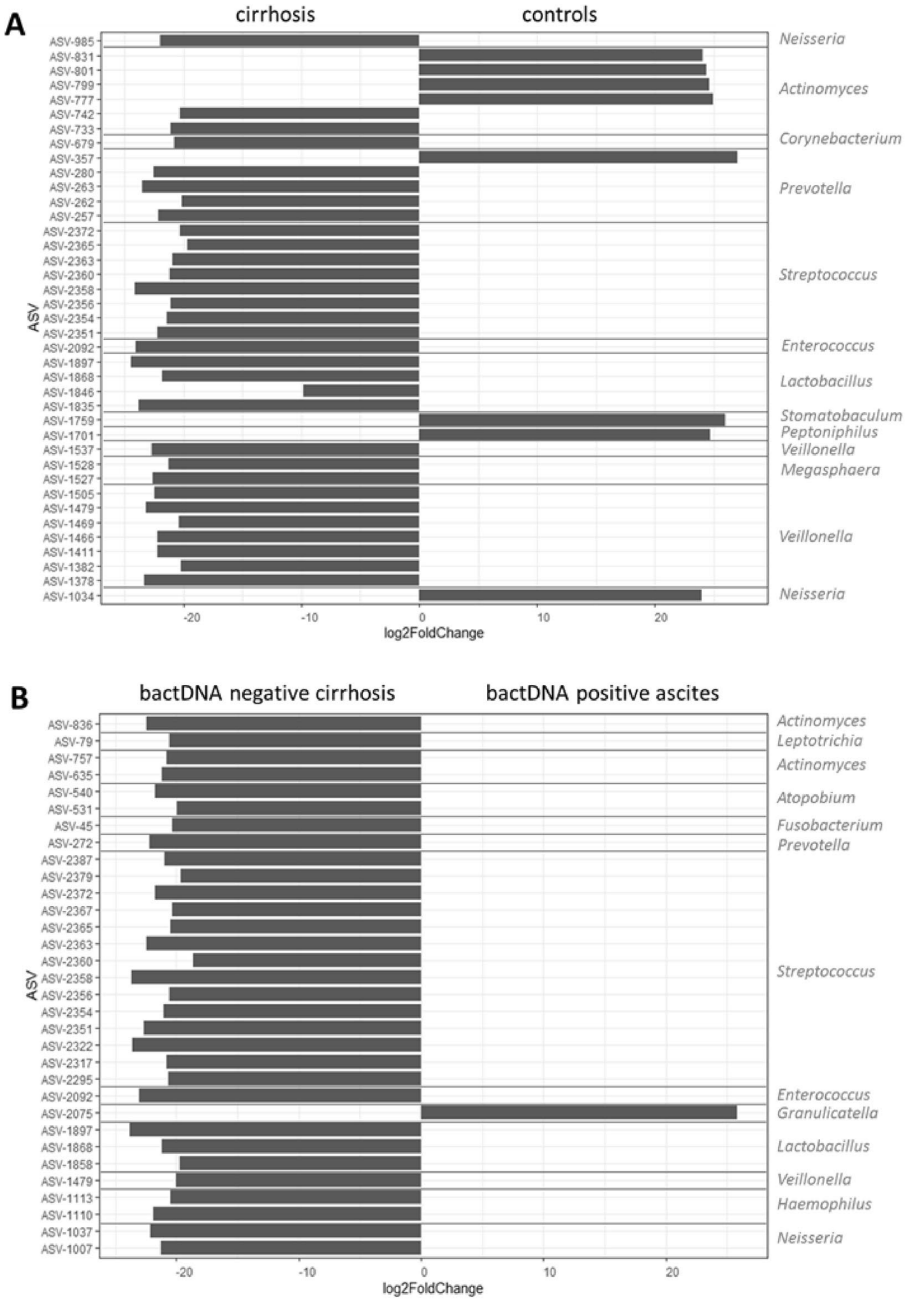
Amplicon sequence variants with significant different abundance in duodenal fluid samples from cirrhosis vs controls (**A**) and duodenal fluid samples from patients with bactDNA negative blood or ascites samples vs patients with bactDNA positive ascites (**B**) displayed as log2-fold change.

The higher bacterial microbiome diversity observed in patients with cirrhosis was a result of a significantly increased proportion of the genera *Prevotella*, *Veillonella, Lactobacillus, Enterococcus* and *Streptococcus* (Fig. 3A). Moreover, patients with bactDNA positive ascites (n = 7) showed a significantly higher abundance of *Granulicatella* in duodenal fluid compared to patients with bactDNA negative samples (Fig. 3B).

The core microbial community (defined as ASVs present in 90% of the samples) of all samples (cirrhosis and control patients) comprised three ASVs. ASV-2071 belonged to the *Granulicatella* genus, ASV-2145 and ASV-2340 to the genus *Streptococcus*. The latter two were the only core ASVs identified from cirrhosis patients. ASV-2071 was present in some cirrhosis samples but did not reach the 90% cut-off that was used for core microbiome definition. In contrast, 10 ASVs were identified from control patients, including six ASVs belonging to *Streptococcus* and one ASV belonging to *Atopobium*, *Veillonella*, *Gemella* and *Granulicatella* each.

## Discussion

This study provides significant insights into the relevance of intestinal bacterial translocation in cirrhosis. Analyses of bactDNA content and its composition in duodenal fluid samples were performed by ultra-clean quantification and subsequent amplicon sequencing of the 16S rRNA gene V3-V4 region. To avoid contamination with free-circulating DNA from nonviable cells we used a DNAse pre-treatment during DNA isolation, hence DNA was extracted only from intact bacterial cells. As major findings, we could show that the amount of bactDNA copies in duodenum of cirrhosis was not different compared to liver healthy controls. However, we identified a distinct and more diverse bacterial spectrum of the duodenum bacterial community in patients with cirrhosis, in particular in patients with bactDNA positive blood or ascites samples. Data strongly suggests a link between intestinal dysbiosis and dissemination of pathogens into blood and ascites rather than simple overgrowth as the source of infectious complications in cirrhosis such as SBP^2,16,17^.

Bacterial composition analyses from polymicrobial samples, e.g. from intestine, is always challenging as the reliable identification and separation of individual bacterial species is the key but has its drawbacks. Large numbers of different techniques which have been used in previous studies to analyze dysbiosis and translocation in cirrhosis, such as cultural techniques or breath testing^18^ differ in terms of yield, accuracy and capacity to distinguish between multiple different pathogens and therefore hamper the comparability with the here presented data.

However, the novelty of our results also lies in the sequence of diagnostic approaches. T-RFLP-analysis was used in a first step as quick analysis tool to monitor changes in the overall duodenal bacterial composition of patients with cirrhosis and controls. There is literature proving its increased sensitivity over culture-based techniques to detect bacterial genera in intestinal samples especially for those with lower population counts^19^. The majority of samples showed a limited number of terminal restriction fragments (T-RF) with elevated relative abundance. These data indicate that the duodenal bacterial community was dominated by only a few bacterial key players.

In mouse models of chronic liver injury induced by steatohepatitis T-RFLP revealed that the composition of gut microbiota was perturbed^20^. To further investigate the duodenal bacterial composition of our patients also with respect to presence or absence of bactDNA in blood or ascites we applied amplicon deep-sequencing of duodenal aspirates. Amplicon sequencing did not show significant differences in Shannon diversity between cirrhosis and controls, which means that comparable numbers of bacterial genera were detected. This corresponds to Chen et al. who were analyzing mucosal duodenal microbiota in cirrhotic patients using high throughput methods^21^. Even the most pre-dominant bacterial genera (*Streptococcus*, *Rothia* and *Veillonella*) presented similar abundances in both groups. In contrast, Santiago et al. who investigated the microbiome of stool specimens detected a higher alpha-diversity in healthy controls compared to cirrhotic patients^22^ whilst other studies reported a significant reduction of microbial diversity in stool samples when decompensated cirrhosis developed^23^. Data comparison and its interpretation are difficult as studies focused on distinct localizations in the intestine.

However, we were able to identify a greater microbial community variability in cirrhosis with respect to the genera *Prevotella*, *Streptococcus*, *Enterococcus*, *Lactobacillus* or *Veillonella* in comparison to controls proving the existence of dysbiosis and expansion of distinct bacterial genera in the duodenum from patients with cirrhosis. Accordingly, previous studies identified cirrhosis-specific microbiota profiles, which were characterized by expansion of gram-positive families of *Enterococcaceae* and *Streptococacceae* and reduction of other families such as *Veillonellacae* etc.^23^. Similar microbial changes where recognised in the saliva of patients with liver cirrhosis and altered intestinal microbiota may play an important role in modulating systemic plasma metabolites in patients with cirrhosis^24^.

The bacterial spectrum in controls was tighter whilst duodenal fluid from cirrhosis showed a broad spectrum of different bacteria. Increasing abundance of intestinal *Enterocooci* was linked to increasing severity of alcoholic liver disease^25^ and abundance of the genus *Streptococcus* was previously associated with leaky gut in cirrhosis^26^ identifying these genera as potential key mediators of liver disease and its complications. We also discovered a significant higher abundance of *Granulicatella* when bactDNA was present in ascites of patients with cirrhosis. However, although the *Granulicatella* genus may be more abundant in duodenum it does not belong to known pathogens linked to the development of SBP^27^. Whether these bacteria might cause infectious complications could not be clarified here. Future analyses have to be performed to understand the association between its higher abundance in duodenal fluid and the presence of bactDNA in ascites and whether *Granulicatella* may alter gut permeability. One study performed in thirty patients with liver cirrhosis identified expansion of *Granulicatella* among others to be associated with the presence of hepatic encephalopathy^28^ and therefore suggest a link between abundance of *Granulicatella* and cirrhosis related complications.

However, if translocating pathogens from the intestine cause systemic bacterial dissemination leading to infectious complications such as SBP it is intriguing why our bactDNA detection rate in blood and ascites was so low. On the one hand, it makes it unlikely that false positive results caused by contaminations played a role in our analyses. On the other hand, this aspect is challenging the assumption that bacteria are permanently present in blood and ascites once bacterial translocation is initiated in cirrhosis. Several factors such bacterial clearance by the innate immunity (e.g. macrophages and neutrophils) and also a potential variability of intestinal barrier function might impact on the presence of viable cells in the systemic circulation. Non-viable cells releasing pathogen associated molecular patterns (PAMPs) might be equally effective in triggering inflammatory reactions through stimulation of pattern recognition receptors macrophages and neutrophils^29^. Further analyses are necessary to understand the individual contribution of above listed bacterial components on the development of cirrhosis related complications^30,31^.

Liver disease severity has been observed to be another substantial confounder for the intensity of dysbiosis and intestinal bacterial translocation in cirrhosis^32,33^ especially during progression from compensated to decompensated cirrhosis and acute-on-chronic liver failure^22,34,35^. However, in contrast to previous duodenal microbiome data which preferably included compensated cirrhosis with Child Pugh A^21^ our patient cohort mainly consisted of end-stage liver disease patients with Child B and C (85.1%) expecting most significant microbial alterations in this advanced liver cirrhosis group^34^. The antibiotic pre-treatment in 30% of cases and also a continuous therapy with lactulose, proton pump inhibitors (PPI) and NSBB might have influenced the presence of bactDNA from viable cells^36,37^. NGS investigation was done to representative duodenal samples of patients with liver cirrhosis (n = 29) and controls (n = 11). Comparing these two groups with focus on NSBB and PPI intake a significant difference could not be detected whereas patients with cirrhosis were treated significantly more frequent with lactulose (S2 Table).

However, there was no significant difference in treatment with NSBB and PPI between the NGS investigation groups of patients with liver cirrhosis (n = 29) and controls (n = 11) whereas the intake of lactulose was significantly higher in patients with cirrhosis (Table 2). Even advanced Redundancy analysis (RDA) showed no significant impact of antibiotic treatment and PPI intake on the duodenal bacterial composition (adjusted R-squared 0.005004818).

**Table 2:** Baseline characteristics of patients whose duodenal fluid was used for 16S rRNA gene amplicon sequencing categorical data are displayed as absolute values and percentage.

Variable	Liver cirrhosis with bactDNA pos blood (n = 7)	Liver cirrhosis with bactDNA pos ascites (n = 7)	Liver cirrhosis with bactDNA neg blood/ascites (n = 15)	All patients with liver cirrhosis (n = 29)	Control patients with bactDNA neg blood (n = 11)	Level of significance (p) cirrhosis vs controls
Age (years), median (range)	50 (40–71)	59 (44–70)	55 (44–66)	55 (40–71)	71 (42–88)	**0.001**
Gender (male), n (%)	5/7 (71.4)	5/7 (71.4)	13/15 (86.7)	23/29 (79.3)	7/11 (63.6)	0.418
BMI (kg m^-2^)	27.1 (23.1–33.2)	23.6 (20.1–38.8)	27.8 (24.4–35.2)	27.7 (20.1–38.8)	25.0 (18.6–32.4)	0.423
MELD score,	12.5 (28–7)	18 (6–37)	17 (8–30)	16 (6–37)	n.a	n.a
Child pugh score, n (%)	A 2/6 (33.3)	A 1/7 (14.3)	A 1/14 (7.1)	A 4/27 (14.8)	n.a	n.a
	B 1/6 (16.7)	B 2/7 (28.6)	B 8/14 (57.1)	B 11/27 (40.7)		
	C 3/6 (50.0)	C 4/7 (57.1)	C 5/14 (35.7)	C 12/27 (44.4)		
SBP, n (%)	1/7 (14.3)	0/7 (0)	1/15 (6.7)	2/29 (6.9)	n.a	n.a
SBP prior sampling (6 months before sampling), n (%)	1/7 (14.3)	2/7 (14.6)	0/15	3/29 (10.3)	n.a	n.a
Antibiotic therapy						
–At baseline	2/7 (28.6)	1/7 (14.3)	5/15 (33.3)	8/29 (27.6)	2/11 (18.2)	0.696
–7 d before baseline, n (%)	3/7 (42.9)	1/7 (14.3)	4/15 (26.7)	8/29 (27.6)	0/11	0.080
PPI, n (%)	5/7 (71.4)	6/7 (85.7)	11/15 (73.3)	22/29 (75.9)	8/11 (72.7)	1
NSBB, n (%)	1/7 (14.3)	1/7 (14.3)	6/15 (40.0)	8/29 (27.6)	0/11	0.080
Lactulose, n (%)	2/7 (28.6)	4/7 (57.1)	10/15 (66.7)	16/29 (55.2)	0/11	**0.003**

Metric data are displayed as either mean ± standard deviation or median (range). All patients with cirrhosis and controls were compared using Mann–Whitney *U* test or Chis-square test.

Significant values are in bold.

It has been described that different segments of the intestine may vary in terms of bacterial composition and the major source of bacterial translocation is still debated. Tremendous work has been done to investigate the role of the colon by analyzing the patient’s fecal microbiome at different stages of liver cirrhosis^38,39^. However, previous studies suggest a high relevance of the small intestine as a source for bacterial translocation in patients with advanced cirrhosis^9^. As shown here the duodenum might be of interest to assess dysbiosis in cirrhosis as it is easily accessible via esophagogastroduodenoscopy and regular bacterial composition surveillance during clinically intended interventions could be an interesting approach helping to identify patients at increased risk for complications of bacterial translocation such as SBP.

Limitations of the study arise from the endoscopic procedure itself and its inherited risk of bacterial contaminations from the upper gastrointestinal tract that might affect the results. In addition, due to ethical reasons the control group did not consist of healthy individuals but of outpatients with an indication for an upper gastrointestinal endoscopy such as gastritis or duodenal ulcer which might also impact on the bacterial composition. In addition, only a small number of patients with cirrhosis had quantifiable bactDNA in ascites so that the relevance of this finding remains uncertain in this particular cohort.

In conclusion, we could show that the duodenal bacterial composition but not the bacterial quantity is altered in patients with liver cirrhosis, especially in those with presence of bactDNA in blood or ascites with higher variability of duodenal microbial composition.

The influence of individual genera on the one hand and bacterial components such as endotoxins and free circulating DNA on development of cirrhosis related complications on the other hand should be assessed in future studies.

## Materials and methods

### Study design

This study included 125 Caucasian individuals admitted to the University Hospital Leipzig between 2011 and 2017 which were scheduled for a clinically indicated esophagogastroduodenoscopy. Overall, 103 patients had a liver cirrhosis (82.4%) and 22 patients without liver disease and infections (17.6%) were chosen as control group. Duodenal fluid samples (n = 125), corresponding blood samples (n = 125) within 5 days and ascites samples within 7 days after endoscopic procedure (n = 39) were collected from each individual (Fig. S5). In 38 out of 125 patients (30.4%) duodenal irrigation with 10 ml water through the endoscop was required to collect duodenal fluid (3 ml). To minimize the risk of cross-contaminations from the upper gastrointestinal tract during the procedure endoscopes were moved quickly into the duodenum without aspiration of fluid during passage through the esophagus and stomach. Moreover, sterile catheters were used to collect duodenal samples. BactDNA results from these samples were normalized for water dilution. Inclusion criteria were the presence of liver cirrhosis and age over 18; intensive-care patients were excluded. The control cohort included liver healthy individuals with indication for diagnostic esophagogastroduodenoscopy due to upper abdominal pain (n = 7), esophagitis/gastritis (n = 5), neoplasia (n = 5), biliary tract disease (n = 4) and gastric ulcer (n = 1).

The following clinical and laboratory parameters were assessed at baseline (date of duodenal fluid collection): etiology of cirrhosis, sex, age, BMI, Model for End-Stage Liver disease (MELD) score, Child–Pugh score, liver-function tests (total Bilirubin, Albumin, level of Alanine aminotransferase and Gamma-glutamyltransferase), white blood cell count (WBC), C-reactive protein (CrP), drug history and history of cirrhosis related complications such as HE, esophageal varices and bleeding. In contrast to European guidelines, the leukocyte count of all ascites samples was used to define SBP as the PMN count was not part of routine diagnostics and therefore not available for all cases (Table 1). One patient with negative leukocyte count but positive cytomorphology and subsequent antibiotic therapy was considered as SBP positive. All data were collected retrospectively.

The study protocol conformed to the ethical guidelines of the 1975 Declaration of Helsinki and was approved by the ethics committee of the University of Leipzig (No. 356-10ek). All patients gave their written informed consent.

### Culture-independent characterization of bacterial DNA

For PCR analyses, 50 ml of ascites were collected and resuspended in 3 ml supernatant after centrifugation (4000 g, 25 min, RT). Three ml of whole blood and 3 ml of duodenal fluid were collected during routine blood collection and esophagogastroduodenoscopy. Samples were stored at − 20 °C with a final concentration of 20% glycerin until processing as described^40^.

For comparison with routine diagnostics 10 ml of ascites were inoculated in aerobic and anaerobic BacT/Alert blood culture bottles (bioMérieux, Marcy l’Étoile, France). BactDNA from blood, ascites and duodenal fluids was extracted using a DNA-free isolation kit (MolYsis Complete5, Molzym, Bremen, Germany) according to the manufacturer’s protocol. A DNase degrading free circulating pathogen (and human) DNA is included in this kit enabling the analyses of DNA from intact cells. BactDNA positive samples were differentiated from bactDNA negative samples by qualitative 16S rRNA gene based PCR and DNA content was subsequently quantified as previously described^41^. Here, we did not consider the different number of 16S rRNA gene operons for each species and thus solely used the copies of the 16S rRNA gene for quantification. The entire process was performed under ultra-clean conditions with DNA-free plastic material and reagents to prevent cross contaminations with other samples or from environment. A negative control for DNA isolation and PCR was used in each analysis. Subsequent sanger sequencing (GATC, Constance, Germany) was applied for the identification of bactDNA in positive ascites and blood samples due to their low proportion of multiple pathogens.

### T-RFLP analysis for the differentiation of bacterial DNA

T-RFLP was used as screening method to identify differences in bacterial community structure in duodenal samples. Bacterial 16S rRNA gene fragments were amplified in a 35 cycle PCR with a 6-FAM labelled reverse primer 797r (Eurogentec, Cologne, Germany) with PCR conditions as previously described^41,42^.

For T-RFLP-profiling, 50 ng of the purified PCR product were digested for 3 h with 1 U of the restriction endonuclease *MspI* (New England Biolabs, Frankfurt, Germany). Length of terminal restriction fragments (T-RF) was determined using the ABI Prism 3100 genetic analyzer (Applied Biosystems, Foster City, USA) and the Genemapper V3.7 software (Applied Biosystems). Clone analyses from duodenal samples (n = 3) was performed with 40 clones each in order to assign the genus type to the most relevant T-RF.

### 16S rRNA gene amplicon sequencing

16S rRNA gene amplicon sequencing was performed for duodenal fluid of patients with either a corresponding bactDNA positive blood sample (n = 7) or a bactDNA positive ascites sample (n = 7). Additionally, exemplary duodenal fluid samples of patients with cirrhosis (n = 15) and of controls (n = 11) based on heterogeneous T-RFLP patterns as shown in Fig. 1 were analyzed. For this sub-analysis, patients were matched according to age, gender, BMI, MELD, antibiotic treatment as well as use of proton pump inhibitors (Table 2).

Amplicon sequencing was performed as described elsewhere^42^. Briefly, a 464-bp long fragment from the 16S rRNA gene V3-V4 region was amplified using primers described by Klindworth et al.^43^. Amplified products were prepared for sequencing using Nextera XT kits (Illumina, San Diego, USA) and were subsequently sequenced with a MiSeq desktop sequencer using MiSeq Reagent Kits v2 500-cycles (Illumina).

### Statistical analysis and sequence processing

Statistical analysis of clinical data and 16S rRNA gene-specific PCR was performed using SPSS 22 software (SPSS Inc., Chicago, IL). For patients’ baseline data categorical variables were displayed as percentages and the continuous variables were displayed as median and range. A two-sided p-value of < 0.05 was considered statistically significant. Group comparison of metric parameters was performed using a Mann–Whitney *U* test and of categorical parameters by Chi-square test. All 16S amplicon deep-sequencing data were analyzed as previously described^44^. Illumina Sequencing data were processed using the QIIME2 pipeline^45^.

Primer sequence clipping and low-quality read trimming were performed using default parameters. Dereplication was done with DADA2^46^. Sequences were binned into amplicon sequence variants (ASVs) at a minimum sequence identity of 97% and they were further processed for chimera removal and taxonomic assignment using the QIIME2 default parameters. Taxonomy classification was performed using the SILVA 128 16S rRNA gene database^47^. ASVs and meta data were imported to R^48^ and mitochondrial, chloroplast and non-bacterial sequences were removed from the dataset. Statistical analyzes of microbiomes were performed using the phyloseq, microbiome^37^ and ampvis2^38^ packages^49^.

### Supplementary Information

Supplementary Information.

## Data Availability

The datasets generated and/or analysed during the current study are available in the NCBI repository (Sequence Read Archive). The project has been registered with the BioProject database under BioProject accession number: PRJNA884282. web link: https://www.ncbi.nlm.nih.gov/sra/PRJNA884282.
