# Supplementary material for: Changes of the bacterial composition in duodenal fluid from patients with liver cirrhosis and molecular bacterascites
Source: Sci Rep. 2023 Dec 27;13:23001. doi: 10.1038/s41598-023-49505-3 (PMC10754895; doi:10.1038/s41598-023-49505-3)
Supplement: Supplementary file 1 — Supplementary Information. [file 41598_2023_49505_MOESM1_ESM.pdf]

Changes of the bacterial composition in duodenal fluid from patients with liver cirrhosis and molecular bacterascites

Jim Höppner[1], Sandra Krohn[1], Ellen H.A. van den Munckhof[2], René Kallies[3], Adam Herber[1], Katharina Zeller[4], Jan Tünnemann[5], Madlen Matz-Soja[1], Antonis Chatzinotas[3], Stephan Böhm[6], Albrecht Hoffmeister[5], Thomas Berg[1*], Cornelius Engelmann[1,7,8*]

Supplementary

Sampling Nr.	Cohort	Result bactDNA in blood	qPCR 16S [copies/ml]
M12-00031	Liver cirrhosis	*Propionibacterium*<br>*Acinetobacter (johnstonii)*	$7{,}34 x10^3$
M12-01816	Liver cirrhosis	*Staphylococcus sp.*	$7{,}88 x10^3$
M12-02938	Liver cirrhosis	*Staphylococcus (epidermidis)*	$4{,}80 x10^3$
M12-03266	Liver cirrhosis	*Acinetobacter sp.*<br>*Staphylococcus sp.*	$5{,}16 x10^3$
M13-01442	Liver cirrhosis	*Staphylococcus (epidermidis)*	$9{,}59 x10^4$
M14-00909	Liver cirrhosis	*Enterococcus (cecorum)*	$1{,}67 x10^5$
M15-00976	Liver cirrhosis	*Pseudomonas sp.*<br>*Aerococcus (viridans)*<br>*Enterococcus (casseliflavus)*	$1{,}14 x10^4$
M12-00154	Control	*Raoultella (planticola)*<br>*Enterococcus sp.*	$2{,}13 x10^4$
M13-00593	Control	*Staphylococcus (lugdunensis/hominis)*	$7{,}65 x10^3$

S1 Table. Results of 16S qPCR showing bacterial species detected in blood.

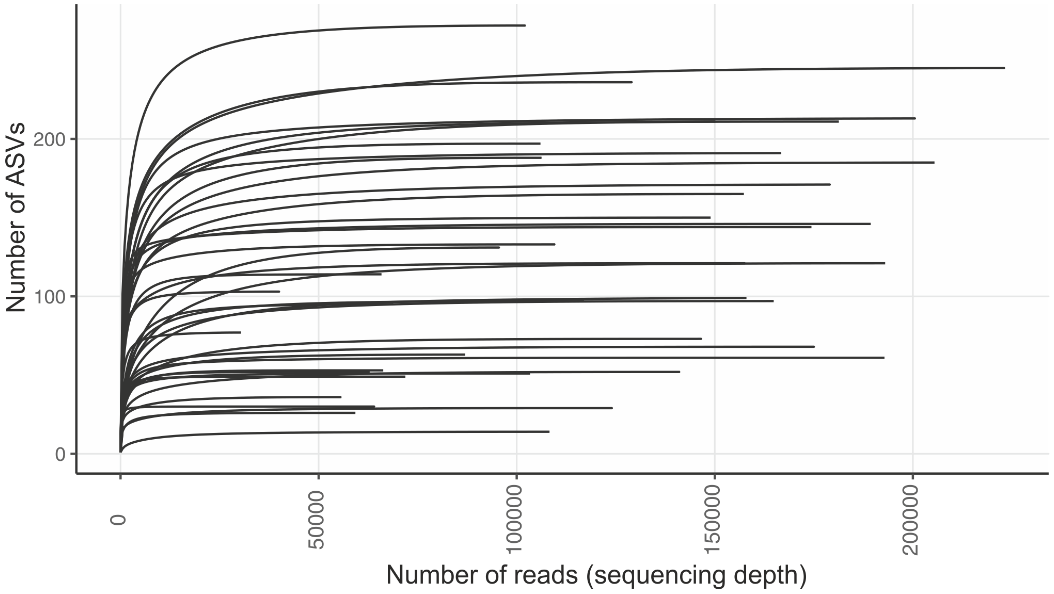

S2 Figure. Rarefaction curve for the 40 duodenal samples that were used for 16S rRNA gene amplicon sequencing. The x axis represents the number of sequences per sample while the y axis represents the species richness (total number of ASVs per sample).

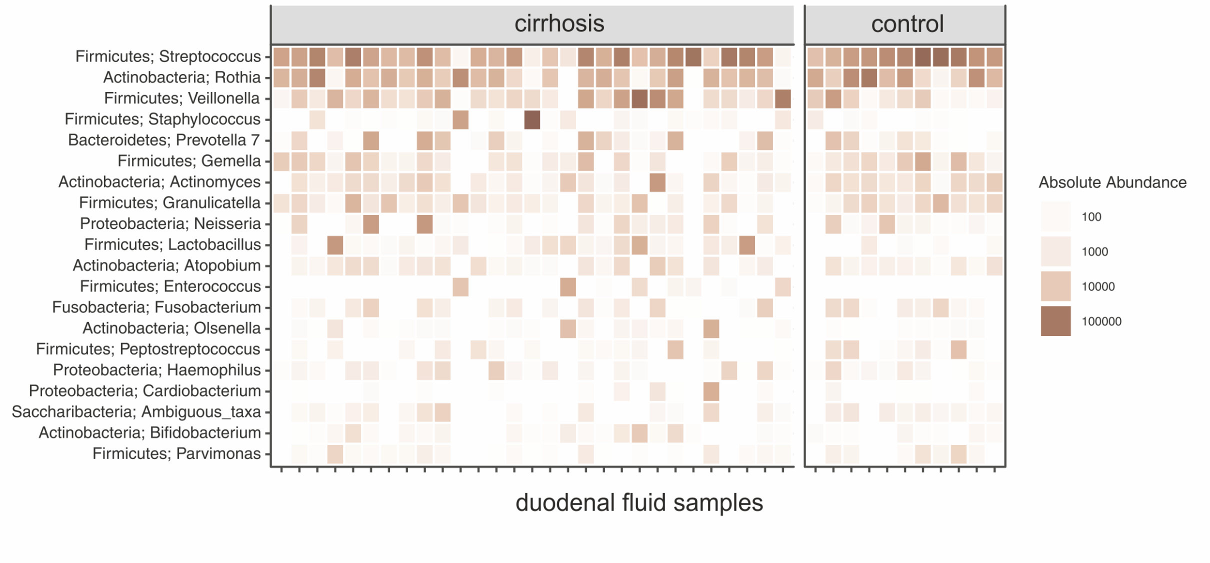

S3 Figure. Heatmap showing the absolute abundance of the 20 most abundant genera per duodenal fluid sample in patients with cirrhosis (n=29, left panel) and controls (n=11, right panel).

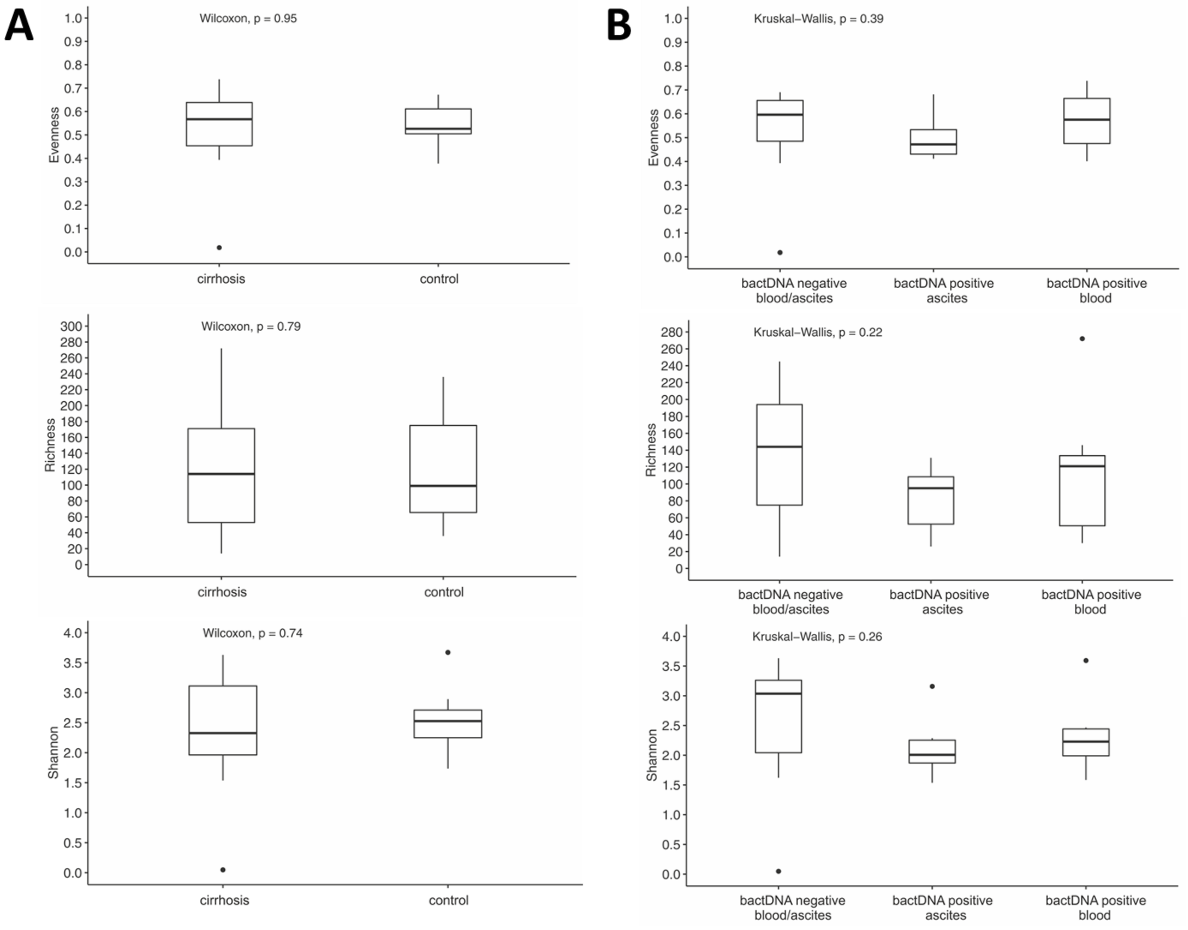

S4 Figure. Alpha-diversity analyses (Pielou's evenness, richness, Shannon diversity) of duodenal samples for different patient cohort: (A) cirrhosis and controls and (B) cirrhosis with negative or positive bactDNA findings in blood and ascites.

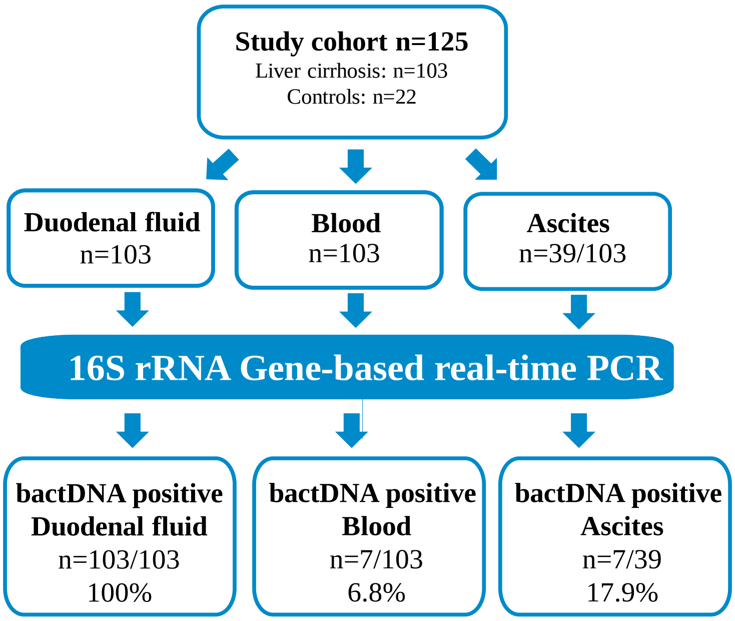

S5 Figure. Study design including 125 patients (103 patients with liver cirrhosis, 22 patients in control group).

	**Liver cirrhosis, n = 103**	**Controls, n = 22**
**Antibiotic therapy at baseline**	n=32<br>Ceftriaxon n=8<br>Piperacillin/Tazobactam n=5<br>Amoxicillin/Clarithromycin n=4<br>Ciprofloxacin n=3<br>Cefuroxim n=2<br>Levofloxacin n=1<br>Sultamicillin n=1<br>Imipenem n=1<br>Vancomycin n=1<br>Rifaximin n=1<br>Clindamycin n=1<br>Ciprofloxacin/Metronidazol n=1<br>Ciprofloxacin/Imipenem n=1<br>Ceftriaxon/Metronidazol n=1<br>Cefotaxim/Metronidazol n=1	n=3<br>Imipenem (Pneumonia) n=1<br>Ampicillin/Sulbactam (urinary infaction) n=1<br>Clarithromycin/Amoxicillin (bacterial gastritis) n=1
**7d before baseline**	n=30<br>Piperacillin/Tazobactam n=7<br>Ceftriaxon n=6<br>Ciprofloxacin n=2<br>Sultamicillin n=2<br>Amoxicillin/Clarithromycin n=2<br>Levofloxacin n=1<br>Cefuroxim n=1<br>Imipenem n=1<br>Rifaximin n=1<br>Ceftriaxon/Metronidazol n=1<br>Ciprofloxacin/Imipenem n=1<br>Piperacillin/Tazobactam/ Ceftriaxon n=1<br>Piperacillin/Tazobactam/ Vancomycin n=1<br>Piperacillin/Tazobactam/ Meropenem n=1<br>Amoxicillin/Clarithromycin/ Ceftriaxon n=1<br>Ciprofloxacin/Metronidazol/ Piperacillin/Tazobactam/ Ceftriaxon n=1	n=0

S6 Table. List of antibiotic therapy in both study cohorts at baseline and 7 days before baseline.

**Primary Disease/Reason for gastroscopy**	**quantity, n=22**
Malignant diseases/screening: screening of gastrointestinal malignancies in patients with metastatic diseases	n=6
Upper abdominal pain with diagnosis of gastroesophageal reflux disease and/or gastritis	n=6
Biliary tract diseases	n=6
Gastric ulcer	n=2
Diagnostic based on pain and weight loss	n=2
Oesophageal obstruction	n=1

S7 Table. Indications for gastroscopy performed in control group.
